# *Helicobacter pylori* infection of AZ-521 cells reveals a type IV secretion defect and VacA-independent CagA phosphorylation

**DOI:** 10.1242/dmm.032813

**Published:** 2017-12-01

**Authors:** Nicole Tegtmeyer, Steffen Backert

**Affiliations:** Friedrich Alexander University Erlangen, Department of Biology, Division of Microbiology, Staudtstr. 5, D-91058 Erlangen, Germany

We refer to the recent publication by Nakano and co-workers in *Disease Models & Mechanisms* (Nakano et al., 2016). This report claims that the *Helicobacter pylori* vacuolating cytotoxin (VacA) is the crucial factor inducing the activation of the host cell kinase for translocated CagA, Src, via a mechanism involving the receptor phosphatase RPTP-α using the human duodenum carcinoma cell line AZ-521 as a novel model system. In fact, VacA and a type IV secretion system (T4SS) with its effector protein CagA represent major virulence determinants of *H. pylori* (Boquet and Ricci, 2012; Foegeding et al., 2016; Naumann et al., 2017)*.* VacA is a paradigm of pore-forming toxins, which contribute to the pathogenesis of peptic ulceration. Several cellular receptors have been described for VacA, including various lipids, sphingomyelin, heparin sulphate, receptor protein tyrosine phosphatase (RPTP)-α, RPTP-β, EGF receptor, fibronectin and integrin β_2_ (CD18) on T cells. Secretion of VacA is associated with the formation of membrane channels, induction of apoptosis and inhibition of immune cell proliferation (Boquet and Ricci, 2012; Foegeding et al., 2016). The second important virulence factor is CagA, encoded by the T4SS in the *cag* pathogenicity island. The T4SS-pilus is induced upon host-cell contact and requires the receptor integrin α_5_β_1_ for the transport of CagA into target cells (Kwok et al., 2007). After delivery, CagA becomes tyrosine-phosphorylated (CagA^PY^) at EPIYA motifs by Src and Abl kinases, and mimics a host cell factor for triggering intracellular signaling cascades affecting cytoskeletal, proliferative, anti-apoptotic and other responses (Mueller et al., 2012; Backert et al., 2015). In particular, it has been demonstrated previously that the activation of Src and CagA phosphorylation proceeds in a T4SS-dependent manner (Stein et al., 2002; Selbach et al., 2003; Kwok et al., 2007), and the purified T4SS pilus-associated protein CagL alone can profoundly stimulate the activation of Src and other tyrosine kinases, via binding to integrin α_5_β_1_, in various gastric and nongastric cell lines (Tegtmeyer et al., 2010).

**Fig. 1.**
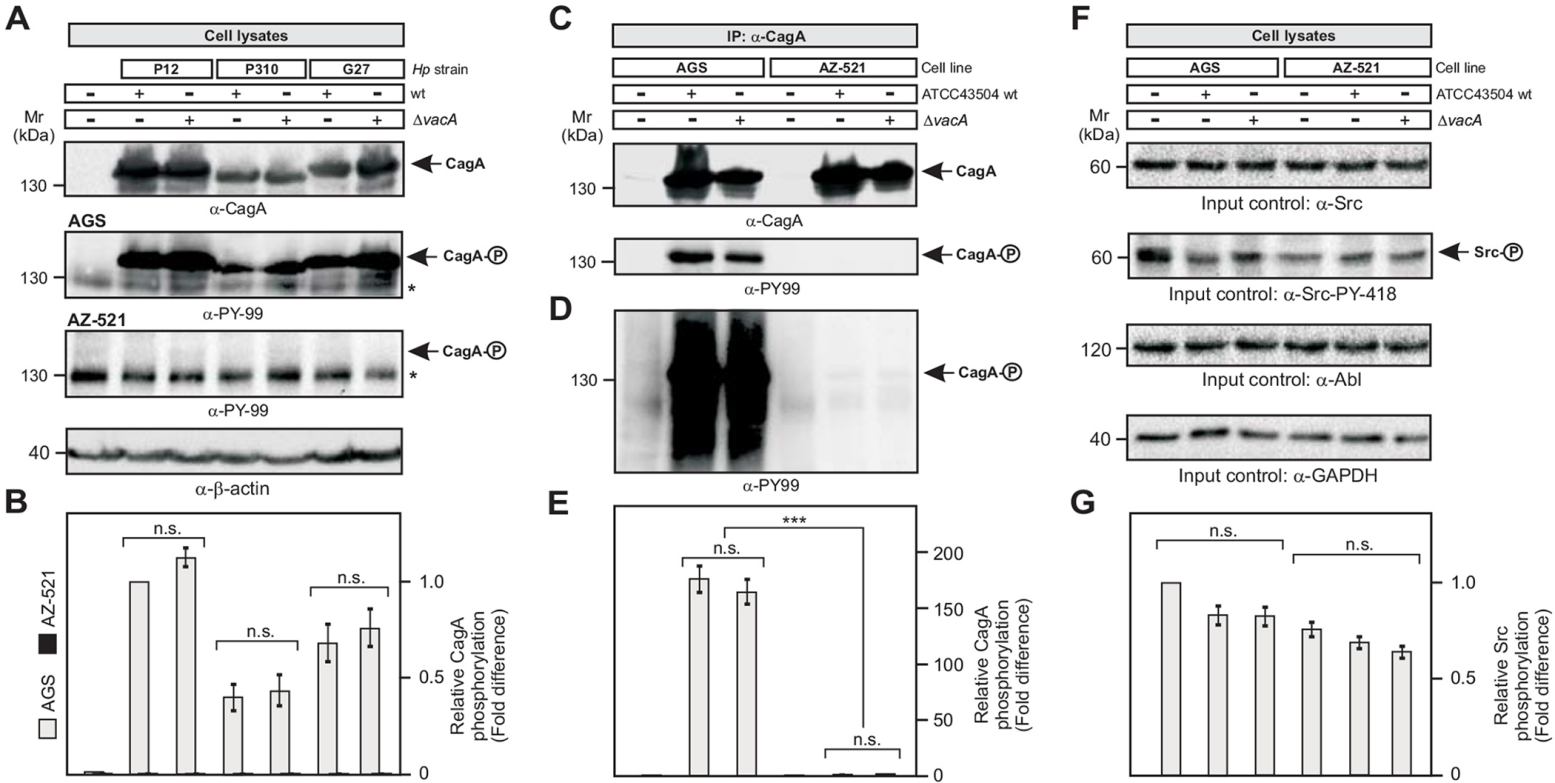
***H. pylori* infection of AZ-521 cells** **reveals very low type IV secretion competence compared with that of AGS cells and VacA-independent CagA phosphorylation.** (A) AGS and AZ-521 epithelial cells were infected with the indicated *H. pylori* wild-type strains and Δ*vacA* mutants for 9 h using an MOI of 100 on six-well plates. Resulting protein lysates were probed with the anti-PY-99 antibody to detect CagA^PY^ as described (Mueller et al., 2012). The anti-CagA and anti-β-actin blots served as loading controls for bacterial and host cell proteins, respectively. Arrows indicate the position of CagA forms on the gels. The asterisks mark a phosphorylated 125 kDa host cell protein migrating below CagA. (B) Quantification of CagA phosphorylation signals in panel A using the luminescence image analyzer (Mueller et al., 2012). The relative CagA phosphorylation levels are shown as fold change. The signal in lane two was set as 1. Data (mean±s.e.m.) are representative of three independent experiments. (C) Immunoprecipitation of CagA from AGS and AZ-521 cells infected with *H. pylori* strain ATCC43504 and an isogenic Δ*vacA* mutant for 9 h on 10-cm petri dishes. The blots were probed with anti-PY-99 and anti-CagA antibodies and exposed for 6.1 s. CagA^PY^ signals were only detected in the AGS cell samples. (D) Exposure of the anti-PY-99 blot for 77 s revealed very strong and very weak CagA^PY^ signals for AGS and AZ-521 cells, respectively (arrow). (E) Quantification of CagA^PY^ signals in panel D. The signal in lane five was set as 1. Data (mean±s.e.m.) are representative of three independent experiments, and show that CagA phosphorylation is more than 160-fold stronger in AGS cells than in AZ-521 cells. (F) Control blots for panels C-E showing the input levels for Src, Abl and GAPDH in lysates from the various infected cell lines. The anti-Src-PY-418 blot reveals the activation status of Src (Selbach et al., 2003; Nakano et al., 2016). (G) Quantification of anti-Src-PY-418 blot signals in F. The signal in lane one was set as 1. Data (mean±s.e.m.) indicate that Src is highly active in both AGS and AZ-521 cells.

To solve the discrepancy between the report by Nakano et al. (2016) and previous studies on the T4SS-dependent activation of Src in various cell lines (Stein et al., 2002; Selbach et al., 2003; Kwok et al., 2007; Tegtmeyer et al., 2010; Mueller et al., 2012), we followed the protocol by the authors and utilized AGS and AZ-521 cell lines from the same origin as described (Nakano et al., 2016). These cells were infected with three different *H. pylori* wild-type strains and isogenic Δ*vacA* deletion mutants under identical conditions for 9 h at a multiplicity of infection (MOI) of 100 (Fig. 1A). The resulting protein lysates were probed with anti-PY-99 and anti-CagA antibodies to visualize the levels of CagA phosphorylation as indicative for its translocation (Kwok et al., 2007). The results show that *H. pylori* can profoundly induce CagA^PY^ for each wild-type and Δ*vacA* strain in AGS cells (Fig. 1A, arrows). Surprisingly, we detected no significant difference in the CagA^PY^ levels of wild-type strains versus those of Δ*vacA* mutants in AGS, and discovered no CagA^PY^ signals at all in infected AZ-521 cells (Fig. 1A,B). This result was confirmed in at least five independent experiments including shorter and longer infection times (data not shown). Next, we infected AGS and AZ-521 cells with strain ATCC43504 as used by the authors followed by immunoprecipitation of CagA. The corresponding blots were probed with anti-CagA antibodies, confirming that equal amounts of CagA proteins were precipitated (Fig. 1C). The anti-PY-99 blot exposed for 6.1 s exhibited strong CagA^PY^ signals in the AGS-infected samples, but not in infected AZ-521 cells (Fig. 1C). However, exposure of this anti-PY-99 blot for 77 s revealed overexposed CagA^PY^ signals in AGS cells and very faint bands for infected AZ-521 cells (Fig. 1D, arrow). Densitometric quantification of the signals revealed that CagA phosphorylation in AGS cells is ∼163-fold to 176-fold higher than that in infected AZ-521 cells, and no significant difference was seen between the CagA^PY^ signals from wild-type and Δ*vacA* mutant *H. pylori* (Fig. 1E). This suggests that either translocation of CagA or the kinase activity of Src is widely diminished in AZ-521 cells. To answer this question, we determined Src activity using an activation-specific antibody for Src phosphorylation at the autophosphorylation site Y-418. The results revealed similar strong phospho-Src signals in both cell lines (Fig. 1F). We noticed a slight reduction in overall Src activity in AZ-521 compared with AGS cells; however, this difference cannot account for the dramatic differences seen in the CagA^PY^ signals between the two cell lines (Fig. 1G). Thus, these results strongly suggest that translocation of CagA into AZ-521 cells is widely impaired compared with that into AGS cells, rather than differences in the activity of Src. We were also unable to detect significant differences in the expression of phosphatidylserine between the two cell lines, and propose that T4SS pilus formation or an imbalanced expression of integrin α_5_ and β_1_ chains, or lack of CEACAM receptors, could be involved in the observed T4SS defect in AZ-521 cells. This should be clarified in future studies.

As a possible explanation for the conflicting data, we assume that incubation of host cells with purified VacA and transfection of CagA as performed by Nakano and co-workers do not reflect the actual situation during infection (Nakano et al., 2016). We also assume that some observations were overinterpreted by the authors. Thus, we think that the proposal to use AZ-521 cells as a new infection model for studying novel mechanisms of type IV secretion and phosphorylation of CagA is highly questionable. Taken together, we provide evidence that AZ-521 cells exhibit a significant defect for the uptake of translocated CagA by the *H. pylori* T4SS, and therefore represent no useful model system to study processes involved in CagA translocation and phosphorylation. However, although the defect of AZ-521 is remarkable and worth investigating further at the molecular level, both AGS and AZ-521 represent nonpolarized cell lines. Instead, more efforts should be directed to clarify in future how CagA can be delivered and phosphorylated in polarized gastric epithelial cell models, which are more close to the situation of *H. pylori* infection *in vivo*.
